# Developing high energy dissipative soliton fiber lasers at 2 micron

**DOI:** 10.1038/srep13680

**Published:** 2015-09-08

**Authors:** Chongyuan Huang, Cong Wang, Wei Shang, Nan Yang, Yulong Tang, Jianqiu Xu

**Affiliations:** 1 Key Laboratory for Laser Plasmas (Ministry of Education) and Department of Physics and Astronomy, Shanghai Jiao Tong University, Shanghai 200240, China; 2 College of Optical Sciences, University of Arizona, Tucson, Arizona 85721, USA; 3 IFSA Collaborative Innovation Center, Shanghai Jiao Tong University, Shanghai 200240, China

## Abstract

While the recent discovered new mode-locking mechanism - dissipative soliton - has successfully improved the pulse energy of 1 μm and 1.5 μm fiber lasers to tens of nanojoules, it is still hard to scale the pulse energy at 2 μm due to the anomalous dispersion of the gain fiber. After analyzing the intracavity pulse dynamics, we propose that the gain fiber should be condensed to short lengths in order to generate high energy pulse at 2 μm. Numerical simulation predicts the existence of stable 2 μm dissipative soliton solutions with pulse energy over 10 nJ, comparable to that achieved in the 1 μm and 1.5 μm regimes. Experimental operation confirms the validity of the proposal. These results will advance our understanding of mode-locked fiber lasers at different wavelengths and lay an important step in achieving high energy ultrafast laser pulses from anomalous dispersion gain media.

Realizing high energy ultrashort pulses at various wavelengths is very important for a variety of scientific and industrial applications and has been the persistent pursuit of scientists. Possessing advantages like compactness, robustness, and good spatial mode quality, fiber lasers have been considered as attractive candidates for generation of ultrashort laser pulses. However, fiber lasers lag well behind their solid-state counterparts in both pulse energy and pulse duration^1^. To improve the performance of fiber lasers, various mode-locking techniques have been proposed and extensively explored^2^,^3^,^4^,^5^,^6^,^7^,^8^,^9^,^10^,^11^,^12^,^13^,^14^,^15^,^16^,^17^,^18^. In this developing process, three kinds of mechanisms have played important roles: dispersion-managed soliton^2^,^3^,^4^, similariton^9^,^10^,^11^,^12^, and dissipative soliton (DS)^13^,^14^,^15^,^16^,^17^,^18^. Among these, the DS mode-locking takes advantage of the balance between not only nonlinearity and dispersion but also gain and loss, giving rise to pulse energies 1 ~ 2 orders of magnitude larger than those from conventional soliton mode-locking^13^,^14^. Up to now, the DS pulse energies from 1 μm and 1.5 μm fiber laser systems have exceeded 20 nJ with femtosecond pulse duration^15^,^16^,^17^.

Recent years, ultrashort laser pulses at 2 μm have attracted considerable interest and have been used in extensive application areas like LIDAR, surgical operation, molecule spectroscopy, remote sensing, and so on^19^,^20^,^21^,^22^. Contrary to the encouraging results of DSs achieved in the 1 μm and 1.5 μm spectral regions, the pulse energies of 2 μm fiber lasers still remain at a low level. This is because the readily available gain fibers (GFs) in the 2 μm region show relatively large anomalous dispersion, which results in mode-locking operation of 2 μm fiber lasers often lying in the conventional soliton regime^23^,^24^,^25^,^26^,^27^,^28^. By inserting elements (such as gratings) with small normal dispersion into the cavity, the performance can be improved to certain extent through dispersion management, but the underlying mechanism puts an ultimate limit on the pulse energy of this kind of fiber laser, i.e. the soliton area theorem^29^.

For the purpose of breaking through the pulse energy bottleneck of 2 μm fiber lasers, some researchers have tried to employ normal dispersion fiber components to achieve DS mode-locking in this spectral region. With a chirped fiber Bragg grating providing normal dispersion, Gumenyuk *et al.* demonstrated 2 μm DSs in a semiconductor saturable absorber mirror (SESAM) mode-locked thulium/holmium fiber laser with pulse energy of 2.2 nJ^30^. However, its relative narrow spectral bandwidth (5 nm) limited the shortest dechirped pulse duration to ~1 ps. It is well known that the dispersion of a fiber can be shifted through adjusting its core diameter and numerical aperture (NA)^31^, thus specially designed fibers can provide normal dispersion at 2 μm. Using a very small core (2.75 μm) and high NA (0.28) dispersion compensating fiber (DCF), Haxsen *et al.* reported a SESAM/nonlinear polarization rotation (NPR) hybrid mode-locked 2-μm fiber laser^32^. Although the output pulse was dechirped to 482 fs, the pulse energy was only 0.67 nJ, comparable to conventional solitons. Q. Q. Wang *et al.* also reported a 2 μm DS fiber laser with a similar DCF to manage the intracavity dispersion and obtained 0.45 nJ, 2.3 ps pulses^33^.

Here, we present a detailed investigation into the intracavity pulsing dynamics of a 2 μm DS mode-locked fiber laser, revealing some important differences between 2 μm DS fiber lasers and their 1 μm and 1.5 μm counterparts. Because of such differences, DSs at 2 μm, though based on the same theory, have been limited to low energy so far. According to theoretical analysis, we propose that the anomalous dispersion GF should be condensed as short as possible to efficiently decouple gain from dispersion and nonlinearity. In this way, the pulse can avoid too much phase accumulation and thereby evolve stably into high energy DSs. Numerical simulations show that over 10 nJ DSs at 2 μm can be realized. Experimentally, a 4.9 nJ DS with 579 fs dechirped pulse duration is achieved from a SESAM mode-locked thulium-doped fiber laser, which has the further potential for higher pulse energies. To the best of our knowledge, this is the highest energy 2 μm DSs with femtoseconds pulse duration from single-mode fiber oscillators.

## Results

### Numerical simulation

**Figure 1:**
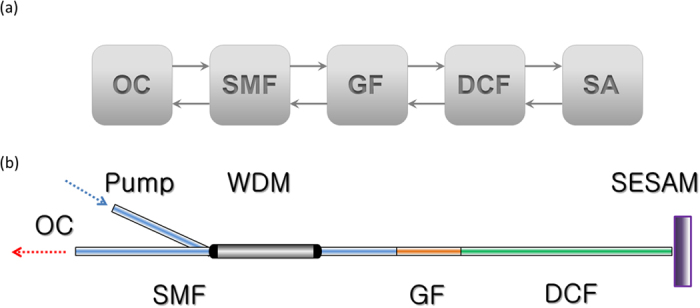
(**a**) Schematic of a 2 μm normal dispersion fiber laser with a linear cavity. DCF, dispersion compensating fiber; GF, gain fiber; SMF, single mode fiber; OC, output coupler; SA, saturable absorber. (**b**) Experimental setup of mode-locked Tm-doped fiber laser.

**Figure 2:**
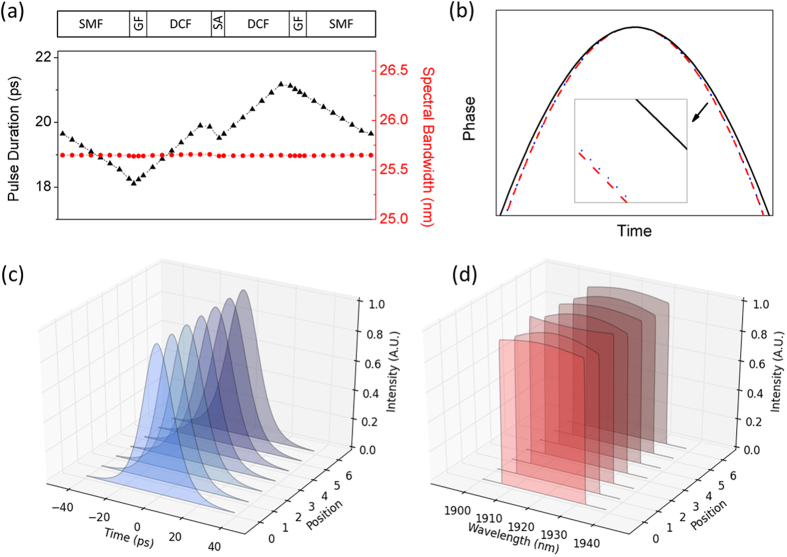
Intracavity Dynamics. (**a**) Pulse duration (black triangles) and spectral bandwidth (red circles) evolution. (**b**) Temporal phase of the solution to the laser plotted after DCF (black solid), after GF (red dashed), after SMF (blue dotted). (**c**) Temporal and (**d**) spectral profiles of the pulse after SMF, GF, DCF, SA, DCF, GF, SMF, respectively.

A schematic model for the mode-locked laser is illustrated in Fig. 1(a). The single mode fiber (SMF), DCF, and GF complete the dispersion map. The GF is a highly thulium doped single mode fiber. Simulation is carried out through solving the nonlinear Schrodinger equation (NLSE). Detailed parameters of all related elements are present in Methods. Start with white noise, the calculation proceeds until a steady state is reached. A typical and stable solution with pulse energy of 5 nJ is obtained when Psat = 3.5 kW and Esat = 3.4 nJ are set, and its pulsing and spectral evolutions are shown in Fig. 2. Owing to the joint effects of normal group velocity dispersion (GVD) and nonlinearity (NL) in DCF, the pulse propagates with its duration increasing monotonically. The weakly broadened pulse is then compressed by the SMF and GF segments with anomalous GVD. The spectral bandwidth of the pulse with steep edges is nearly constant during its circling in the cavity. However, the spectrum top exhibits sharp peaks near the edges after the pulse is amplified in the GF and then undergoes self-phase modulation in the followed DCF. Then the spectrum is reshaped by SA, DCF, GF, and SMF, successively, and evolves back to the near-flat top shape. Figure 2(b) shows the temporal phase shape evolution in the three kinds of fibers (DCF, GF and SMF). We see that there is only a small amount of phase shift induced by the short GF, which is effectively compensated by the other two kinds of fibers (DCF and SMF).

**Figure 3:**
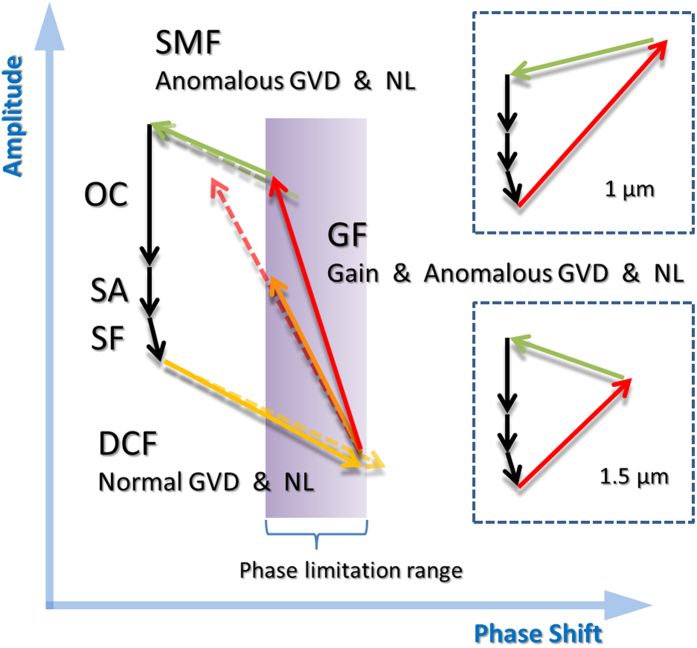
Qualitative illustration of the amplitude and phase balances in a DS fiber laser at 2 μm, along with the 1 μm and 1.5 μm counterparts (insets). The red, green, and black arrows in the insets share the same meaning with the main figure.

To gain deeper insight into the intracavity pulsing dynamics, a qualitative illustration for 2 μm DSs is summarized in Fig. 3, along with their 1 μm and 1.5 μm counterparts (insets)^34^. In the 1 μm and 1.5 μm spectral regions, the GFs (Yb-doped or Er-doped) have normal dispersion and introduce positive phase shift, which can be compensated by the negative phase shift provided by the SMF, even if the shift is relatively large (see the insets of Fig. 3). However, the scenario is quite different in the 2 μm wavelength regime, where the GFs (Tm-doped or Tm-Ho-codoped) provide anomalous dispersion and thus negative phase shift. To realize 2 μm ultrafast DSs, both DCF and SMF are necessary for dispersion management, and the net normal dispersion should not be too large. For a given 2 μm fiber laser cavity, the tolerance of phase shift left for the GF (Fig. 3) is limited to a relatively small phase limitation range (purple area). By increasing fiber length, the phase shift incurred by the DCF or SMF is small (yellow or green dashed arrow) while that induced by the GF is significant (red dashed arrow). The shorter GF (red arrow) has a larger slope and hence can achieve higher pulse energy within the phase limitation range. On the contrary, the longer GF (orange arrow), due to its smaller slope, has to sacrifice a large part of amplitude (and energy accordingly) to reduce its phase shift under the tolerable level. Therefore, pulses from a cavity with a longer GF have lower energies in this spectral region.

Based on the above analysis, we propose using a condensed GF (shortened to a small length while provides adequate gain at the same time) to scale the pulse energy of DSs in the 2 μm and mid-infrared spectral regions, where the dispersion of gain media are usually anomalous. Under this condition, the GF will have a large slope (Fig. 3), and thus can provide high enough pulse energy while keep the phase accumulation within the limitation range.

**Figure 4:**
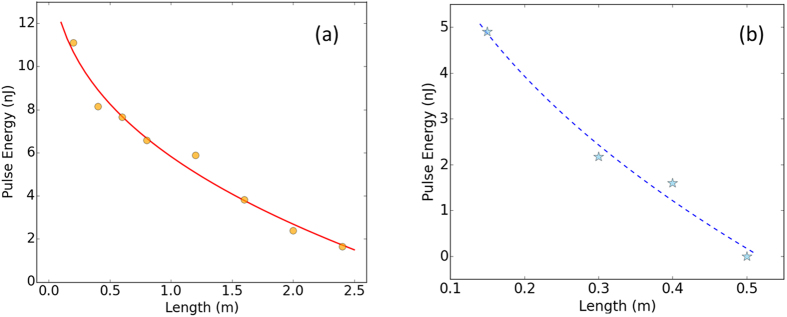
(**a**) Calculated (circle dots) and (**b**) measured (asterisk dots) maximum pulse energy versus the length of GF under the pump power of 1 W. The curves are exponential fittings.

To confirm the idea, simulations are also performed in the above mode-locked fiber laser (Fig. 1) to observe the GF length dependence of the pulse energy. The simulated maximum output pulse energies with various GF lengths are indicated in Fig. 4(a). It is clear that decreasing the GF length will dramatically increase the pulse energy. For instance, as high as 11 nJ pulses are supported by the cavity with a 0.2 m GF, and the pulse can be further dechirped to ~100 fs outside the cavity. This confirms our expectation for achieving high energy ultrashort pulses with anomalous dispersion GFs: shortening the anomalous dispersion GF to a sufficiently small length can significantly improve the DS pulse energy.

### Experimental operation

Encouraged by the simulation results, a highly doped thulium fiber is shortened to different lengths but with a net normal cavity dispersion to experimentally realize high energy DS mode-locking at 2 μm. Experimental setup for the mode-locked fiber oscillator with a linear cavity is schematically shown in Fig. 1(b). Considering the trade-off between enough gain and less phase shift, short lengths of single-cladding Tm-doped fiber (tens of centimeters) are chosen to decouple gain from GVD and NL. The simple design of the cavity offers great flexibility for future modifications or integrating with other function systems. Under stable mode-locking operation, the maximum pulse energies with different GF lengths are shown in Fig. 4(b). It is clear that the experimental results follow the trend of the simulation prediction, i.e. the pulse energy increases quickly as the length of GF decreases. When the GF length is shortened to ~15 cm, the pulse energy is close to 5 nJ, and detailed laser characteristics are shown as the following.

**Figure 5:**
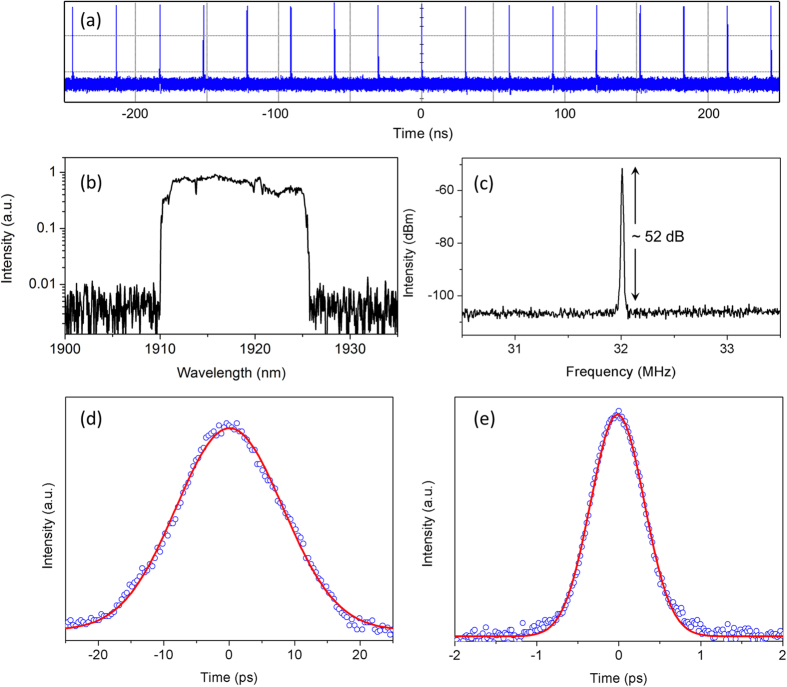
(**a**) Pulse train, (**b**) laser spectrum, and (**c**) RF spectrum of the mode-locked Tm-doped fiber laser. The autocorrelation traces of the pulse (**d**) before and (**e**) after compression, respectively.

With the 15 cm GF, the laser reaches its continuous wave (CW) threshold at ~500 mW of pump power. When pump power is increased to ~650 mW, stable CW mode-locking is self-started. Remarkably, no Q-switching or Q-switched mode-locking is observed. This is due to the large output coupling ratio, which suppresses the intermediate transitions between the CW laser operation and the CW mode-locking regime^35^. The stable CW mode-locked operation maintains when pump power is further increased up to the maximum value. With the maximum available pump power of 1 W, average output power of 158 mW for the 2 μm DS is obtained. Figure 5(a) displays the oscilloscope trace of the mode-locked pulse train at the maximum output, showing a repetition rate of ~32 MHz (corresponding to the round-trip time of ~31 ns). Consequently, the maximum single pulse energy is ~4.9 nJ. The laser spectrum (at the highest output) is analyzed by a mid-infrared spectrum analyzer with a resolution of 0.1 nm, as shown in Fig. 5(b). The wavelength is centered at 1918 nm with a 3-dB spectral width of 15 nm. Steep spectral edges indicate the typical characteristics of DSs^13^,^14^. The radio-frequency (RF) spectrum has a ~52 dB signal-to-noise ratio (Fig. 5(c)). Figure 5(d) shows the autocorrelation trace of the pulses directly produced from the cavity at the maximum output. Assuming a Gaussian shape, it corresponds to pulse duration of 16 ps. The time-bandwidth product is calculated to be 18, which is a large departure from the Fourier transform limited value. The highly chirped pulse is dechirped to 579 fs (Fig. 5(e)) outside cavity by coupling into a ~25 m SMF-28 fiber. After compression, the time-bandwidth product of the laser pulse is reduced to 0.7.

## Discussion

According to simulation results, to achieve high energy DSs at 2 μm, the GF length should be as short as possible to keep phase shift within the phase limitation range while provides enough gain at the same time. To this end, highly doped GFs (short length with enough gain) should be adopted to complete the cavity. Such kind of GF can decouple gain from dispersion and suppress phase shift. This can efficiently avoid the pulses’ evolution to conventional solitons during amplification in the GF. In addition to 2 μm, mid-infrared fiber lasers (usually with anomalously dispersive gain media) can also utilize the same strategy to scale DS energy. It should be noted that this short GF proposal is valid for both linear and ring laser cavities. In a ring cavity, the pulse passes every element only once during one cycle, leading to less phase shift accumulation. Consequently, a larger phase limitation range and higher pulse energy can be expected from ring-cavity mode-locking. However, the drawback is introducing more bulk optical components (such as filter), which may compromise the system robustness.

Guided by the theoretical model, we experimentally demonstrate a net normal dispersion mode-locked Tm-doped fiber laser with a linear cavity. The laser delivers 4.9 nJ DSs with pulse duration of 579 fs after dechirped. To the best of our knowledge, this is the highest energy for 2 μm femtosecond pulses directly generated from single-mode fiber oscillators, and more than twice the previously achieved pulse energy^30^. In fact, the laser has the potential to produce higher pulse energies (>10 nJ), comparable to those from the 1 μm and 1.5 μm counterparts. Simulation shows that pulses from the same cavity can be improved up to 11 nJ if pump power is increased, and even higher pulse energies are achievable with further cavity parameters optimization. In experimental operation, the mode-locking state is very stable and there is no evidence of pulse splitting or multi-pulsing within our available pump power. Therefore, the output pulse energy of the current system is simply limited by the pump power. For further energy scaling of 2 μm DSs, the 1.5 μm pump laser should be amplified to a higher power level with an Er/Yb fiber amplifier. Due to lower cost, smaller size, and higher power, laser diode at 793 nm is also a good pump option if double cladding condensed GFs are available. On the other hand, more condensed GFs should be adopted if their doping level can be further improved. The recently reported highly Tm-doped silicate glass fibers^26^ (with a gain of >2 dB/cm) make this route feasible. Therefore, with higher pump power, more condensed GFs, and further optimizing parameters, ultrafast 2 μm pulses with energy exceeding 20 nJ is very probable in the near future.

## Methods

Numerical simulations are based on the well-known nonlinear Schrodinger equation with gain^9^,^13^,^14^


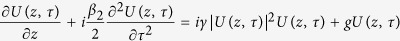


where *U*(*z*, *τ*) is the envelope of the field, z is the propagation coordinate, and 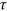 is the time delay parameter. The SMF (8.2/125 μm, 0.14 NA) has a length of 1.4 m, with β_2_ = −67 ps^2^/km and γ = 0.001 (Wm)^−1^, while the DCF (2.2/125 μm, 0.35 NA) is 1.5 m long with β_2_ = 93 ps^2^/km and γ = 0.007 (Wm)^−1^, respectively. The 0.2 m GF (5/125 μm, 0.24 NA), with β_2_ = −12 ps^2^/km and γ = 0.003 (Wm)^−1^, has the gain g = g_0_/[1 + E_pulse_/E_sat_ + (ω − ω_0_)^2^/Δω^2^], where g_0_ corresponds to ~30 dB of small-signal gain, E_sat_ is the gain saturation energy, and the gain bandwidth is assumed to be 90 nm. The saturable absorber (SA) is simulated by the transfer function T = 1 − l_0_/[1 + P(τ)/P_sat_], where the unsaturated loss l_0_ is set to 0.7, P(τ) is the instantaneous pulse power, and P_sat_ is the saturation power. A spectral filter (SF) with 150 nm bandwidth is inserted to describe the bandwidth of SA. The equations are solved with the split-step Fourier method.

Experimentally, the pump source is a CW erbium/ytterbium-codoped fiber laser with maximum output of 1 W centered at 1550 nm. The pump light is delivered into the gain fiber (with absorption of ~1.2 dB/cm at 1550 nm) by a 1550/2000 nm wavelength division multiplexing couplers (WDM), which is made by SMF-28 fiber. The coupling efficiency is ~95% with splice loss included. The parameters of these three kinds of fibers are consistent with the values used in the simulation. Consequently, the total net normal dispersion of the cavity is ~0.04 ps^2^. On the output end, the perpendicularly cleaved fiber facet (~4% Fresnel reflection) is employed to provide laser feedback and acts as the output coupler. In this way, most of the intracavity energy can be extracted. The other end of the cavity fiber is directly butt coupled to the resonant Sb-based SESAM with a reflectance of ~85% at 1900 nm, a modulation depth of ~25%, and a saturation fluence of ~35 mJ/cm^2^. The laser output power is measured with a power meter (Thorlabs) and the laser spectrum is recorded with a spectrum analyzer (Bristol). A 2.5 GHz Agilent oscillator with a 12.5 GHz EOT photodetector and an APE autocorrelator are used to characterize the laser pulsing dynamics.

## Additional Information

**How to cite this article**: Huang, C. *et al.* Developing high energy dissipative soliton fiber lasers at 2 micron. *Sci. Rep.* **5**, 13680; doi: 10.1038/srep13680 (2015).
